# Language and Mathematics Performance: A Systematic Umbrella Review and Meta-Meta-Analysis

**DOI:** 10.3390/jintelligence14080152

**Published:** 2026-07-23

**Authors:** Mehmet Ertürk Geçici, Fidan Geçici, Metin Kaya

**Affiliations:** 1 Department of Mathematics Education, Faculty of Education, Afyon Kocatepe University, 03200 Afyonkarahisar, Türkiye; 2 Department of Turkish Language Education, Faculty of Education, Afyon Kocatepe University, 03200 Afyonkarahisar, Türkiye; fugur@aku.edu.tr; 3 Department of Educational Sciences, Faculty of Education, Istanbul Medipol University, 34810 Istanbul, Türkiye

**Keywords:** language, mathematics, reading, meta-meta-analysis, umbrella review

## Abstract

With the aim of determining the relationship between language and mathematics performance using a more general approach, this study examines the findings of primary meta-analyses using the meta-meta-analysis method. To achieve this, the study synthesized 12 independent primary meta-analyses representing an aggregated sample of more than 3.6 million participants. Because some primary studies overlapped across reviews, this figure reflects the cumulative rather than the unique number of participants. The results suggest a positive, moderate, and statistically significant relationship between language and mathematics performance (*ES* = 0.51, *z* = 19.20, *p* < .001). To explain the heterogeneity identified among the studies, the study further conducted moderator analysis on various categorical and continuous variables. The findings show that meta-analyses that focus on a specific language skill have larger effect sizes than studies that deal with language in general. The study also determined that the effect sizes differed statistically significantly across the language subgroups. A striking finding is that the relationship of reading, listening, writing, and vocabulary skills with mathematics performance is stronger than that of phonological processing skills. In conclusion, the study highlights the need to redesign mathematics instruction holistically, incorporating practices such as reading/listening comprehension, vocabulary development, and the effective use of mathematical language.

## 1. Introduction

A main focus of research in mathematics education is identifying factors related to students’ mathematics achievement (Amland et al., 2025; Breit et al., 2025). Current systematic reviews and meta-analysis reveal that mathematics achievement is shaped by the interaction among numerous variables, including cognitive, affective, and environmental factors (Abín et al., 2020; Wang et al., 2023). One of these variables, the relationship between language and mathematics achievement, has recently attracted growing scholarly interest (Chen et al., 2025; De Keersmaeker et al., 2024; Susperreguy et al., 2024). Mathematics is often considered unrelated to language, yet mathematical thinking is largely supported by linguistic processes, given the connection between language and thought (Peng et al., 2020). Indeed, skills such as reading comprehension in problem solving (Akın, 2022; Lerkkanen et al., 2005), vocabulary in the teaching of mathematical concepts (Ünal et al., 2021), or listening comprehension when reading a mathematical text (Lu et al., 2022), involve an interaction between language, thinking, and mathematics. Therefore, identifying the magnitude of the relationship between language and mathematics and understanding the underlying mechanisms are critical both for improving the quality of mathematical practices in the classroom and for making decisions that collectively evaluate these two disciplines together within educational policies.

Due to their multifaceted nature, language and mathematics have been studied together from various theoretical and empirical perspectives. In fact, research has explored the neuroscientific aspects of these two disciplines (Pollack & Ashby, 2018; Ünal et al., 2025), connection between multilingualism (Sharma & Sharma, 2023) or second language and mathematics (Nucette et al., 2025), the effect of linguistic interventions on mathematics learning (Arizmendi et al., 2021; Graham et al., 2020), and the affective (Wan et al., 2021) or cognitive (Jung, 2025; Lerkkanen et al., 2005; Susperreguy et al., 2024) relationship between these two. Notably, among these studies, the cognitive relationship between language and mathematics has been widely studied (Peng et al., 2020). Their relationship is more often examined in relation to language sub-skills such as reading (Singer & Strasser, 2017; Ünal et al., 2023), writing (Kim et al., 2024), listening (Moussa-Inaty et al., 2020), vocabulary (Lin et al., 2021; Susperreguy et al., 2024), or phonological processing (Koponen et al., 2017; Yang et al., 2022). In the relevant literature, research aiming at explaining this relationship has incorporated many different moderator variables, including the educational level of the sample (Kim et al., 2024; Lu et al., 2022), the developmental levels of the sample (Daucourt et al., 2020; Jung, 2025; Stevens et al., 2024), and the linguistic features of spoken language (Bergqvist et al., 2018; Lu et al., 2022). The need to analyze these studies has been accompanied by a growing body of first-order meta-analysis (FOM) studies on the relationship between language and mathematics performance (Amland et al., 2025; Kim et al., 2024; Lin et al., 2021; Lu et al., 2022; Peng et al., 2020; Ünal et al., 2023). However, the fragmented nature of the literature limits the generalizability of findings on the relationship between language and mathematics performance. This makes it particularly challenging to compare findings on the relationship of language skills with mathematics performance.

Synthesizing the findings of the first-order meta-analyses is a prerequisite for determining the relationship between language and mathematics performance with a broader approach. However, some meta-analyses findings are inconsistent with each other (e.g., Lu et al., 2022; Singer & Strasser, 2017; Ulum & Küçükdanacı, 2024; Yang et al., 2022). Identifying the reasons for inconsistencies and contradictions is also important. All of these contribute to the need to explore the relationship between language and mathematics performance in a more reliable and generalizable way. This calls for a meta-meta-analysis to synthesize findings from current FOM studies. Acknowledging this, the aim of this study is to examine the relationship between students’ language and mathematics performance. Nevertheless, this study goes beyond merely examining the relationship between language and mathematics performance as a correlation and presents an umbrella review that attempts to demonstrate the relationship between specific language skills, such as reading and writing, and mathematical skills, based on FOM studies. Accordingly, this study seeks to answer the following questions:

1. Is there an overall correlation between students’ language and mathematics performance?
  1.1. Does the overall correlation between students’ language and mathematics performance differ by categorical moderator variables?
  1.2. Do continuous moderator variables predict the relationship between language and mathematics performance?
2. Does the relationship between students’ language and mathematics performance differ by subgroups?

### 1.1. Literature Review

In the present umbrella review, mathematics performance is conceptualized as an overarching construct representing students’ achievement across a broad range of mathematical outcomes as operationalized in the included first-order meta-analyses. These outcomes encompass procedural knowledge (e.g., arithmetic computation and execution of mathematical procedures), conceptual knowledge (e.g., understanding mathematical concepts and relationships), mathematical problem solving, reasoning, and overall mathematics achievement measured through standardized assessments, curriculum-based tests, or researcher-developed instruments (Hiebert & Lefevre, 1986; Kilpatrick et al., 2001; National Council of Teachers of Mathematics, 2000). Because the present umbrella review synthesizes first-order meta-analyses rather than primary studies, mathematics performance was defined according to the operational definitions adopted in the original meta-analyses rather than by imposing a new classification (Amland et al., 2025; Kim et al., 2024; Lin et al., 2021; Lu et al., 2022; Peng et al., 2020). Given this broad conceptualization, understanding the relationship between language and mathematics performance requires examining the cognitive mechanisms through which language supports mathematical learning and performance.

The relationship between language and mathematics performance should be viewed as encompassing the full range of cognitive processes, symbolic systems, and ways in which information is processed. The main underlying reason for this is that the language of mathematics is multidimensional, consisting of linguistic, symbolic, and visual resources (O’Halloran, 2008). For example, to solve a given speed problem using a graph, a student would need to process the visual information in the graph, the symbols in the equation, and the concepts in the language simultaneously (Peng & Lin, 2019). One may also explain this through the Dual Coding Theory. This theory posits that the simultaneous use of verbal and visual representations supports deeper learning and contributes to the permanent processing of information (Paivio, 1986). During these cognitive processes, working memory capacity is crucial for processing linguistic and mathematical information. Working memory is a limited-capacity system that enables the temporary storage and processing of information, and plays a critical role, particularly in complex comprehension and problem-solving processes (Hart et al., 2025; Zhou et al., 2025). Thus, language is a fundamental component for the effective functioning of working memory in mathematics performance, and linguistic development significantly affects the efficiency of working memory in mathematical tasks (Peng & Lin, 2019). In light of this, the relationship between language and mathematics can be understood not only through the direct transfer of skills but also through cognitive mechanisms such as working memory. Accordingly, working memory was discussed as a theoretical mechanism underlying the relationship between language and mathematics rather than as a separate moderator because it was not consistently operationalized across the included first-order meta-analyses and does not constitute a language construct within the conceptual scope of the present umbrella review. Previous first-order meta-analyses have also shown that the relationship between language and mathematics may vary across specific language subskills (Lu et al., 2022; Peng et al., 2020). Therefore, this study examines the relationship between mathematics performance and various language skills in greater detail, and these language skills serve as the potential moderators of the present study. Although executive functioning, attention, motivation, and self-efficacy are well-established correlates of mathematics performance, they were not included as analytical categories because the purpose of this umbrella review was specifically to synthesize evidence concerning language-related constructs rather than broader cognitive or affective predictors of mathematics performance. These variables are acknowledged as important contextual factors but fall outside the conceptual scope of the present review.

#### 1.1.1. Reading and Mathematics

Reading is a complex process that reflects the interaction between cognition and language, encompassing both general cognitive processes and language-specific abilities (Freed et al., 2017). In this respect, reading is a multidimensional language skill that includes word recognition, language comprehension, and active self-regulation processes, as well as “bridging processes” such as reading fluency, vocabulary, morphological awareness, and letter–sound–meaning flexibility, which are located in the common area of these (Duke & Cartwright, 2021). This multidimensional structure is also directly reflected in the studies included in this umbrella review. The studies analyze reading through various components such as decoding (Singer & Strasser, 2017), reading accuracy and fluency (Lu et al., 2022; Ünal et al., 2023), readability (He, 2016), broad reading (Ünal et al., 2023), and reading comprehension (Akın, 2022; He, 2016; Lu et al., 2022; Singer & Strasser, 2017; Ulum & Küçükdanacı, 2024; Ünal et al., 2023). However, it is evident that in a significant portion of the FOM studies, the core dimension of reading examined in relation to mathematical achievement is “reading comprehension”. Since learning mathematics requires students to understand written problems, identify relevant information, and establish meaningful connections between these pieces of information to form coherent mental representations, reading comprehension is one of the fundamental language skills closely related to mathematical performance (Boonen et al., 2016; Sury & Pilchin, 2025). This is because students must deploy both cognitive resources and language skills to understand problems, process information, and generate solutions (Bergqvist et al., 2018). Indeed, various studies have reported a relationship between reading comprehension and mathematics achievement (Boonen et al., 2016; Korhonen et al., 2012; Lerkkanen et al., 2005; Pongsakdi et al., 2020; Sury & Pilchin, 2025). There have also been studies comparing the effects of different languages on reading comprehension and mathematics performance (Bergqvist et al., 2018) and examining the relationship between reading comprehension and mathematics achievement among students whose native language is different from the language of instruction (Greisen et al., 2021). Such a relationship arises from the shared cognitive and linguistic skills in both areas, including text comprehension (Koponen et al., 2007; Lin & Powell, 2022). For this reason, the role of language in the relationship between reading and mathematics is two-fold. Firstly, with each language having different features, language is a predictor of reading comprehension and mathematical achievement on its own. Secondly, language is a key variable that predicts mathematics achievement in relation to reading comprehension.

#### 1.1.2. Writing and Mathematics

Mathematics and writing entail mental processes such as coding, storage, and recall of visual and verbal information (Kim et al., 2024). From a learning perspective, these two areas reveal that mathematics learning, through constructivist and sociocultural methods, is a process in which meaning is actively constructed through the interaction between an individual’s existing knowledge structures and new experiences (Cobb, 1994; Sfard, 2008; Von Glasersfeld, 1995). Writing is a cognitive activity that enables students to adapt and restructure their mathematical knowledge (Idris, 2009). Hence, writing serves as not only a communication tool but also as a cognitive “comprehension” tool for internalizing mathematical concepts (Van Dijk et al., 2023). Moreover, scholarly research emphasizes that writing about mathematics is an activity that can influence the learning process (Thomas et al., 2015). The link between writing and mathematics achievement has been confirmed by several scholarly studies (Davidse et al., 2014; Powell & Hebert, 2016; Pugalee, 2004). Research indicates that the ability to write in a mathematical context serves as a cognitive predictor, facilitating the structuring of mental processes and the internalization of mathematical knowledge and skills.

#### 1.1.3. Listening and Mathematics

Like reading, listening is also a comprehension skill. Listening comprehension is defined as the ability to understand spoken language at the discourse level, and this ability also involves the processes of inferring and constructing meaning (Kim & Pilcher, 2016). Reading comprehension and listening comprehension are not fundamentally the same skills. While reading relies on making sense of written language, listening requires the real-time processing of spoken discourse (Babayiğit & Shapiro, 2020). However, there are also common language components that support both of these skills. For example, vocabulary can be viewed as a fundamental subskill for both skills, as it supports the comprehension process by playing a role in both the formation of background knowledge and the process of making inferences (Wolf et al., 2019). In the context of mathematics lessons, listening involves not only understanding the teacher’s explanations (Planas & Pimm, 2024) but also being able to participate in the mathematical discourse within the classroom (Planas & Pimm, 2024; Schmidt et al., 2024; Sjöblom & Meaney, 2021), following different solution strategies (Mok et al., 2022), evaluating others’ mathematical reasoning (Hintz & Tyson, 2015; Sjöblom & Meaney, 2021), and structuring one’s own thinking within this discourse (Sjöblom & Meaney, 2021). In light of this, verbal comprehension skills are fundamental for effectively supporting mathematics education. Chow and Ekholm (2019) argue that students with solid syntactic perception ability tend to perform better at processing mathematical inputs. Also, various studies in the literature (Fuchs et al., 2015; Lin et al., 2021; Lu et al., 2022; Peng et al., 2020) demonstrate that students’ listening skills are integral to their mathematical competence. Further, Moussa-Inaty et al. (2020) show that reading and listening positively correlate with mathematics when presented simultaneously. Likewise, Lerkkanen et al. (2005) underline that the relationship between reading and mathematics performance, particularly at the first-grade level, is influenced by students’ language comprehension and ability to follow verbal instructions, and state that math success also entails listening comprehension skills, given that math tests are applied orally. However, other findings by Moussa-Inaty et al. (2020) show that only listening skills have a negative correlation with math performance. This is explained by the loss of information before it can be processed adequately in working memory due to the transient nature of auditory input. An individual’s mathematics performance may be negatively affected by the transient nature of auditory information, as they are unable to adequately internalize it.

#### 1.1.4. Vocabulary and Mathematics

Students’ abilities to make sense of mathematical concepts, solve verbal problems, and express abstract ideas also depend on their academic language proficiency (Powell et al., 2017; Ünal et al., 2021). As such, vocabulary constitutes one of the fundamental components of academic language proficiency. A proper understanding of concepts and accurate depiction of problem situations, particularly in a mathematical context, entails knowledge of the meanings of the terms involved. Mathematical vocabulary enables students to internalize the language of mathematics and correctly interpret linguistic expectations in texts (Peng & Lin, 2019; Powell et al., 2017; Ünal et al., 2021). However, while vocabulary supports the comprehension of mathematical language, it is not the same as the ability to comprehend through reading or listening. Vocabulary refers to knowledge of word meanings, but it also represents broader comprehension processes that require integrating this knowledge with the processing of what is read and heard at the syntactic, inferential, and discourse levels (Duke & Cartwright, 2021; Peng et al., 2020). For this reason, although vocabulary supports both reading and listening comprehension, it is a distinct component of language that represents knowledge of word meanings. Accordingly, vocabulary was treated as a distinct language construct in the present umbrella review because the included first-order meta-analyses conceptualized and analyzed it independently from reading and listening (Lin et al., 2021; Lu et al., 2022; Peng et al., 2020). In addition, vocabulary helps reduce misunderstandings by supporting the correct interpretation of concepts encountered in math tasks. Research provides evidence for a correlation between mathematical achievement and overall vocabulary (Chi-San Ho et al., 2025; Peng et al., 2020; Ünal et al., 2021) and mathematical vocabulary (Peng & Lin, 2019; Powell et al., 2017; Stevens et al., 2024; Susperreguy et al., 2024; Ünal et al., 2021). Thus, both overall vocabulary and mathematical vocabulary are variables that affect mathematics performance. Nevertheless, the literature also includes studies reporting conflicting findings (Hornburg et al., 2024).

#### 1.1.5. Phonological Processing and Mathematics

Phonological processing is based on the use of the sound structure of language to process written and spoken information (Peng et al., 2020; Yang et al., 2022). In FOM research, phonological processing involves phoneme awareness (Amland et al., 2025), rapid automatic naming (RAN) (Koponen et al., 2017; Lu et al., 2022; Peng et al., 2020; Yang et al., 2022), phonological awareness (Lu et al., 2022; Peng et al., 2020; Yang et al., 2022), and phonological memory (Lu et al., 2022; Yang et al., 2022). Although phonological processing, decoding, and word recognition are considered related concepts (Waldmann & Levlin, 2024), FOM studies have not included these concepts within the scope of phonological processing. In fact, Singer and Strasser (2017) examined decoding as a component of reading ability. When investigating the literature in the context of the relationship between language and mathematics performance, there are numerous studies investigating the relationship between phonological processing skills and mathematics achievement (Chen et al., 2025; De Smedt et al., 2010; Hornburg et al., 2024; Lyu et al., 2024; Peng et al., 2020; Whitehead et al., 2024; Yang et al., 2022). This link has been explained through three fundamental cognitive mechanisms in the relevant literature. First, it is reported that phonological processing skills play a role in recalling mathematically based information from memory (De Smedt et al., 2010; Lyu et al., 2024; Peng et al., 2020; Yang et al., 2022). Second, phonological processing processes contribute to the encoding and representation of numbers in the mind (Chen et al., 2025; De Smedt et al., 2010; Lyu et al., 2024; Yang et al., 2022). Third, language, incorporating phonological processing, shares a common cognitive mechanism with mathematics (Lyu et al., 2024). In addition, the link between phonological awareness and mathematics performance appears to be bidirectional and may involve a reciprocal interaction (Chen et al., 2025) Still, some findings in the literature suggest that the link between phonological processing skills and mathematics performance may be more limited than that with other language skills (Lu et al., 2022; Peng et al., 2020). Taken together, these research findings suggest that the relationship between phonological processing processes with mathematics performance arises through mechanisms such as coding, storing, recalling, and cognitive processing.

### 1.2. Other Potential Moderators

The strength of the relationship between language and mathematics performance can vary depending not only on fundamental cognitive mechanisms but also on contextual and developmental factors. Therefore, in determining the moderator variables, this study considers both previous meta-analyses’ findings (Lu et al., 2022; Peng et al., 2020; Yang et al., 2022) and theoretical approaches that explain the interaction among language, cognition, and mathematics.

#### 1.2.1. Subfield of Mathematics

As noted above, the relationship between various language skills and mathematics may differ, as may the relationship between language and subfields of mathematics. Indeed, arithmetic operations rely more on numerical processing and automated information, while algebra and especially verbal problems require more intensive linguistic analysis, symbolic interpretation, and integration of multiple representations. This implies that the effect of language skills on mathematics performance may vary across the subfields of mathematics (Akın, 2022). As such, the strength of the relationship between language and mathematics is expected to vary from one subfield of mathematics to another.

#### 1.2.2. Educational Level

As studies show (He, 2016; Ulum & Küçükdanacı, 2024), educational level or student age might influence the strength of the relationship between language and mathematics. While early childhood mathematics learning is predominantly shaped by language-based processes, such as interpreting problem texts; yet, as children grow, symbolic processing and abstract thinking skills become more prominent. Recognizing this, the study incorporates education level as a potential moderator variable to determine whether these differences are significant.

#### 1.2.3. Students’ Developmental Status

Previous meta-analyses have examined whether the relationship between language and mathematics differs according to participants’ developmental status (Akın, 2022; Peng et al., 2020), as cognitive processes may operate differently across these populations. For example, typically developing children may rely on coordinated language and working memory processes to support mathematical learning, whereas this coordination may be disrupted in individuals with learning-related or neurological conditions (Swanson, 2026). Therefore, developmental status was included as a potential moderator in the present study. Following the classifications reported in the included first-order meta-analyses, participants identified as typically developing were coded as typical, whereas those reported as having developmental, learning-related, or neurological conditions (e.g., learning disabilities, attention deficit hyperactivity disorder, hearing impairment, traumatic brain injuries, etc.) were coded as atypical (Akın, 2022; Daucourt et al., 2020; Peng et al., 2020). The present umbrella review adopted the classifications reported in the original first-order meta-analyses and did not introduce or modify any diagnostic categories.

## 2. Methods

### 2.1. Research Design

FOMs refer to meta-analyses that synthesize results from primary studies. This study drew on the meta-meta-analysis method (Eisend & Tarrahi, 2016) to synthesize the findings from FOM studies examining the relationship between language and mathematics performance. The FOM studies on language and mathematics performance were compiled in accordance with PRISMA 2020 guidelines (Page et al., 2021). The PRISMA 2020 Checklist used in this study is presented in Table S1: Prisma checklist. Also, the review protocol was prospectively registered on the Open Science Framework (OSF).

### 2.2. Search Strategies

For the purposes of the present study, articles published in international databases such as SCOPUS, WOS, and EbscoHOST (ERIC, H.W. Wilson, Academic Search Ultimate, etc.) were reviewed. To minimize publication bias, the grey literature was also reviewed through the ProQuest database. Search queries were generated with Boolean operators and abbreviation symbols to include different variations in key structures. When selecting the keywords, the subfields of mathematics and language, as well as some concepts related to these subfields, were also added to the search array, which enabled a more thorough screening. The search string was structured as follows: “The first section covers mathematical concepts, the second section covers linguistic concepts, and the final section contains vocabulary emphasizing meta-analytic studies.” This search string was performed separately for each concept, with the terms searched in the title and abstract fields. Besides this search, Google Scholar, another source of grey literature, was also used to find articles, theses, and reports by searching up to page 35 with the keywords “mathematics”, “language”, and “meta-analysis”. However, no new studies have been identified apart from the ones obtained from the mentioned databases. No year restriction was applied, and the final search was conducted on 27 February 2026. Also, the bibliographies of potential FOM studies recorded were reviewed. No other FOM studies were found during the bibliography search. Supplementary S1 presents details of the strategies used for keywords and searches.

### 2.3. Inclusion and Exclusion Criteria

The FOMs included in the study were systematically evaluated based on established inclusion and exclusion criteria. These criteria were developed by researchers using the existing literature on the relationship between language and mathematics. Table 1 provides the details of these criteria.

### 2.4. Study Selection and Data Extraction

**Figure 1:**
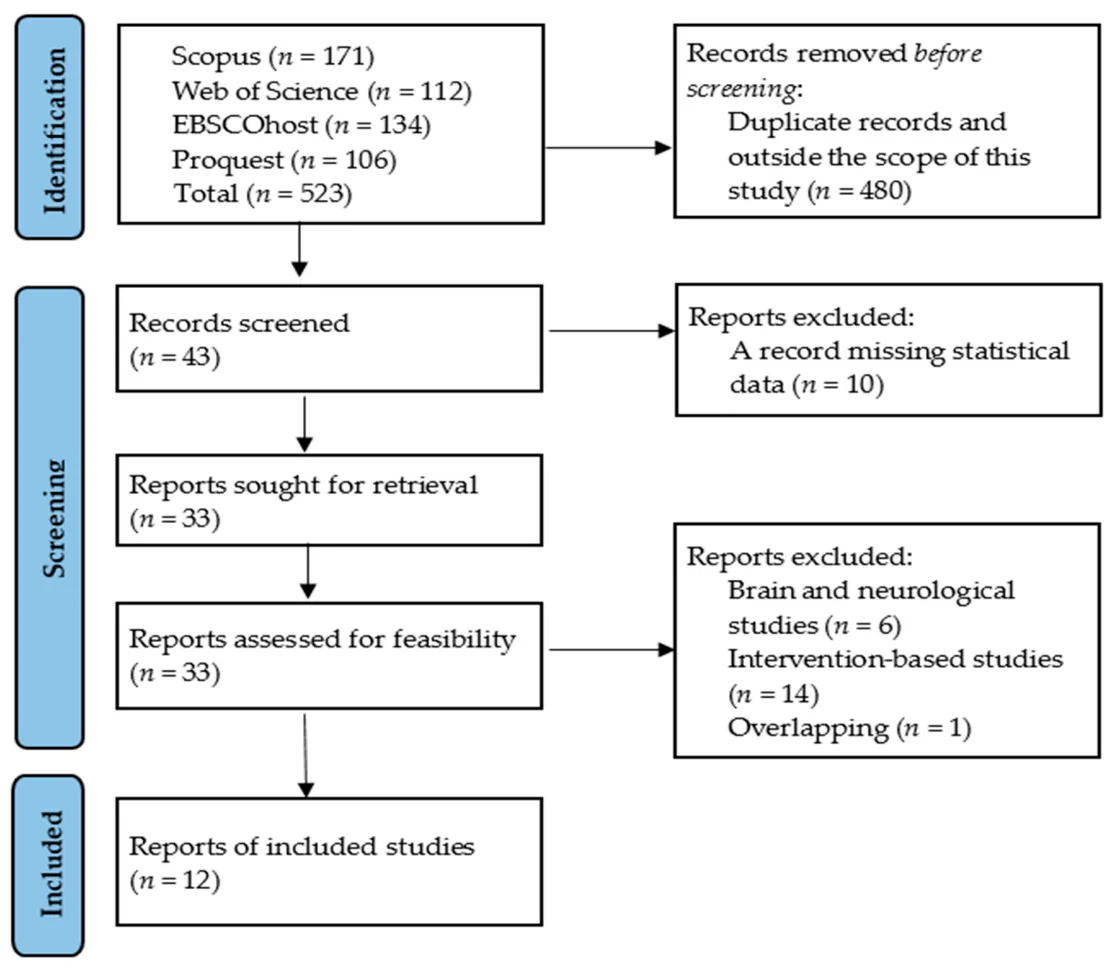
PRISMA flow diagram.

The researchers conducted detailed database searches using specific keywords to identify studies for potential inclusion in this meta-analysis. In the first search, the research titles were reviewed against the inclusion criteria, and then the abstracts were reviewed. In the first stage, most studies that appeared across multiple databases, that did not involve students, that did not focus on language- and mathematics-related cognitive performance, and that focused on second-language or multilingualism concepts related to language and mathematics were excluded. Bilingual and multilingual samples were deliberately excluded to reduce conceptual heterogeneity and provide a clearer interpretation of language performance within a monolingual context. In the next stage, the full texts of the selected studies were examined, and studies that lacked the statistical data required for meta-meta-analysis (*n* = 10) were excluded. Following that, studies not related to educational sciences and aimed at brain and neurological sciences (*n* = 6), and studies involving interventions in the relationship between language and mathematics (*n* = 14), were excluded. Throughout this entire process, the titles, abstracts, and full texts of the studies were reviewed by two independent researchers. Additionally, these two researchers independently extracted data. The remaining 13 studies were included for further analysis. Figure 1 shows the PRISMA flow diagram for the process.

Overlap analysis was applied to the remaining 13 studies that met the inclusion criteria. In other words, the researchers identified how many of the studies analyzed through the existing FOMs in the literature are the same. After the overlap analysis, one of the studies was left out of the meta-meta-analysis. Further information on this is available under the overlapping heading. Consequently, a total of 12 FOMs were included in this study for meta-meta-analysis.

### 2.5. Data Coding

All the researchers collaborated to develop the coding scheme that reflects the characteristics of FOMs. Categories such as Performance type, Participant type of FOM, Education level, Language domain, Math domain, Report type of FOM, Report year of FOM, Location, Quality level for FOM, Number of participants in FOM, Number of primary study in FOM, Effect size number combined in FOM, Publication year range in FOM, and Publication bias status were predetermined. Regarding the coding process, the first three FOMs were coded by all researchers based on the timeliness ranking of the FOMs that comprise this study’s dataset. Then, the two researchers independently coded the entire data set. A high level of agreement was achieved between the independent coders (Cohen’s *κ* = 0.94). All the researchers discussed the inconsistent codes among themselves to reach a final decision. Supplementary S2 includes the potential categories and subcategories of the FOMs.

### 2.6. Quality Appraisal

The JBI Critical Appraisal Checklist (JBICAC) was used to evaluate the quality of the FOMs. The tool consists of 11 items, which are coded as “Yes, No, Unclear, Not applicable” (Aromataris et al., 2015). The quality score for the FOMs was presented as a percentage. The range from 100% to 80% is regarded as high quality, 79% to 60% as medium, and 59% and below as low. The dataset for this study consists of FOMs with high (*n* = 8) and medium (*n* = 4) quality. The lowest percentage of quality is 68.18% and the highest is 100%. And, the average percentage of quality is 88.63% (*SD* = 11.71). This translates to that the overall quality level of the dataset in this study is high. Supplementary S3 provides the detailed quality assessment results for the FOMs.

### 2.7. Assessment of Overlapping

For a reliable effect-size calculation, an overlapping analysis is recommended for meta-meta-analysis (Oh, 2020). The researchers first compiled a list of the research included by the FOMs in the dataset of this study. Then, they performed an overlapping analysis using the Graphical Representation of Overlap for OVErviews (GROOVE) macro. CCA levels were classified as low (<5%), moderate (5–10%), high (10–15%), and very high (≥15%) (Pérez-Bracchiglione et al., 2022). The total number of primary studies derived from meta-analysis in this study is 939, while the number of unique studies is 608. A low level of overall overlap (*CCA* = 2.29%) was observed in the dataset. In a later stage, FOMs with high and very high overlap were examined. This analysis yielded a high overlap (10.4%) between the study by Yang et al. (2022) and the study by Peng et al. (2020), and a very high overlap (17.6%) between the study by Koponen et al. (2017) and the study by Yang et al. (2022). Since the study by Peng et al. (2020) has the broadest scope among these studies, it was included in the meta-analysis. As the study by Yang et al. (2022), which had the narrowest scope, was excluded, there was no longer a very high overlap with the study by Koponen et al. (2017). Supplementary S4 provides the GROOVE graph for the overlapping analysis. Three of the FOMs included in the analysis lacked a list of the studies reviewed; therefore, an overlapping analysis could not be performed on them. However, taking into account the levels of overlap between the FOMs, a sensitivity analysis was conducted.

### 2.8. Data Analysis

*Statistical model and effect size calculation:* Considering the diversity of FOMs, the researchers conducted statistical analysis using a random-effects model (Maitra, 2025). Most of the FOMs in the data set (*n* = 11) reported the Pearson correlation coefficient (*r*), while only one of them (*n* = 1) reported Fisher’s (*Fz*) value. This necessitated converting the effect sizes from all FOMs in the dataset into a common effect size. Pearson’s *r*, when it falls between −1 and +1, results in a reduction in variance (Borenstein et al., 2021). For this reason, the researchers used Fisher’s *z* value as the common metric. They converted the effect sizes generated by the FOMs into *Fz* values and performed statistical analysis with these values. Following that, the researchers converted the *Fz* values back to *r* values for an easier evaluation and interpretation. To assess and interpret effect sizes, the Fisher z-transformed values of the Pearson correlation ranges proposed by Cohen (1988) were used: weak relationship *r* = 0.10 ⟶ *Fz* = 0.10; moderate relationship *r* = 0.30 ⟶ *Fz* = 0.30; strong relationship *r* = 0.50 ⟶ *Fz* = 0.55.

*Unit of analysis:* Sirin (2005) reports that when selecting a unit of analysis for meta-analysis, effect sizes can be considered by their level of aggregation. To minimize potential information loss in meta-analysis, the researchers determined the units of analysis at different levels, considering the research question. For the first research question, the overall effect size values were used as the unit of analysis. The researchers favored this unit of analysis because it allows for a general assessment of the FOMs. For the second research question, effect sizes from a subgroup of FOMs were used as the unit of analysis. This unit of analysis helped identify the missing data. To elaborate, overall effect sizes represent estimates of the general effect size, but some specific data are lost during the calculation. For example, while the overall effect size provides data for all educational levels, independent data for levels such as primary school, secondary school, high school, and university are lost. Recognizing this, the researchers used the independent effect sizes within the FOMs as the unit of analysis to assess the subgroup-level data.

*Moderator and heterogeneity analysis of the dataset:* The overall effect size was computed for the first research problem. The researchers performed Cochran’s *Q* test to estimate the overall heterogeneity. To interpret heterogeneity arising from inter-study and sample-specific factors in the FOMs, they examined *tau*^2^ statistics. Also, to determine the levels of heterogeneity in effect sizes, the advantages and disadvantages of statistical bias analysis and *I*^2^ tests were considered (Alexander, 2020). A Funnel plot, Egger’s Regression test, and Duval and Tweedie Trim and Fill (DTTF) tests were performed, and their results were interpreted together. The effect sizes for the sub-research questions were calculated based on the categorical moderators. The researchers conducted Cochran’s *Q* between-groups test *Q*(b) to determine whether effect sizes differ by categorical variables. A meta-regression test for continuous variables was conducted to assess the extent to which they predicted effect sizes. The researchers explained the heterogeneity based on test results from categorical and continuous moderator variables. For the second research problem, effect sizes were calculated by subcategory, and the *Q*(b) test was applied to assess whether the effect sizes differed statistically across subcategories; lastly, the researchers discussed the contribution of subcategories to heterogeneity.

## 3. Results

**Figure 2:**
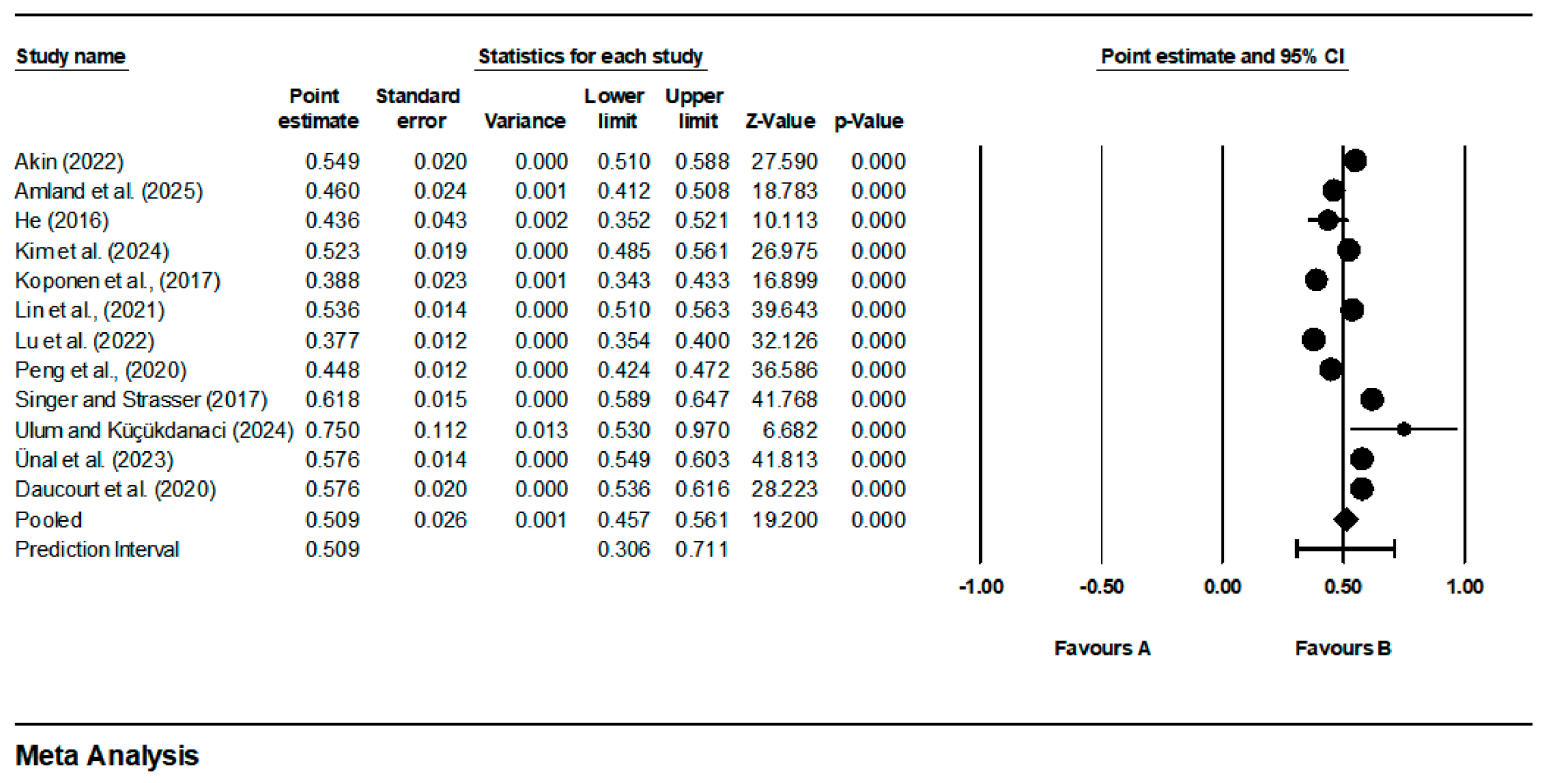
Forest plot. (Akın, 2022; Amland et al., 2025; Daucourt et al., 2020; He, 2016; Kim et al., 2024; Koponen et al., 2017; Lin et al., 2021; Lu et al., 2022; Peng et al., 2020; Singer & Strasser, 2017; Ulum & Küçükdanacı, 2024; Ünal et al., 2023).

The effect sizes in the overall dataset range from a minimum of *ES* = 0.38 to a maximum of *ES* = 0.75. The overall effect size (*ES* = 0.51, 95% *CI* [0.46, 0.56]) was found to be positive and moderate. This finding is also statistically significant (*z* = 19.20, *p* < .001). Figure 2 includes the forest plot showing the distribution of the effect sizes of the FOMs.

**Figure 3:**
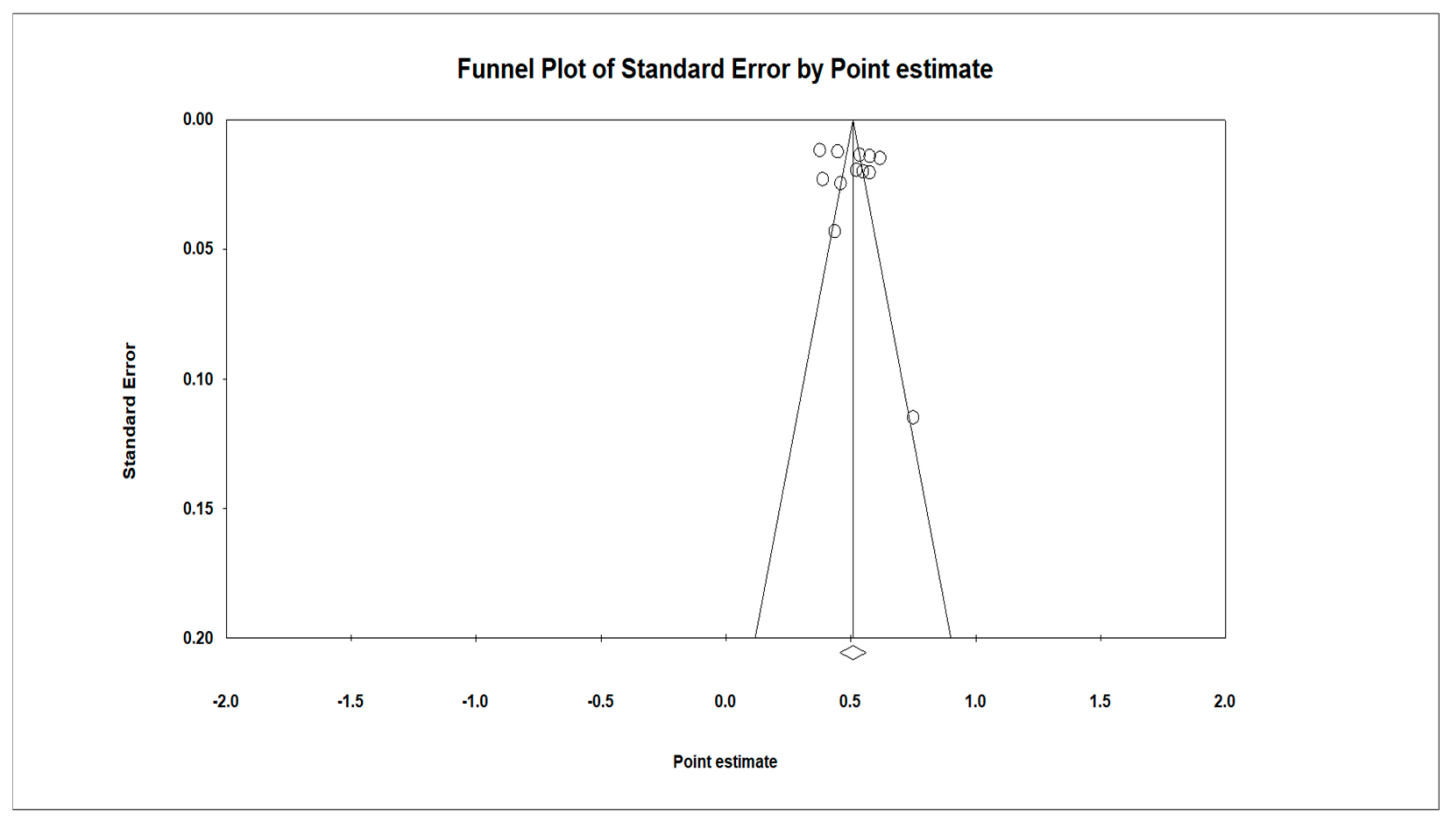
Funnel plot.

It was calculated that the distribution of effect sizes in the included FOM studies was heterogeneous and that this heterogeneity was statistically significant (*Q*(11) = 283.97, *p* < .001). On the other hand, it was observed that heterogeneity was very high (*I*^2^ = 96.13) and that the variance among FOM studies (*tau*^2^ = 0.008) contributed significantly to this heterogeneity. In other words, the effect sizes of the FOM studies differ from one another. In this context, a moderator analysis was conducted to examine the sources of heterogeneity. A Q-between test (*Q*(b)) was performed for categorical moderator variables, and a meta-regression analysis was conducted for continuous moderator variables. Figure 3 shows the funnel plot for the distribution of effect sizes.

The funnel plot for the distribution of effect sizes demonstrates that most effect sizes near the center were distributed symmetrically, but the significant effect size from the FOMs with high standard error in the lower right corner was asymmetrical. No publication bias was detected because the results of the Egger test were not significant (*intercept* = 2.003, *SE* = 3.98, *t* = 0.50, *p* = .63), and the DTTF test indicated that the number of studies that should be added is 0. Considering the publication bias tests together, it can be concluded that the level of publication bias is negligible.

Table 2 presents the categorical moderator and heterogeneity analysis for the overall data set.

The effect sizes of FOMs differed statistically significantly in the language domain (*Q*(b) = 10.05, *p* < .05). Indeed, the specific language domain FOM group (*ES* = 0.55, 95% *CI* [0.50, 0.59]) produced a greater effect size than the undifferentiated FOM group (*ES* = 0.43, 95% *CI* [0.37, 0.49]). The specific language domain FOM group was highly heterogeneous (*I*^2^ = 91.47). For the specific language domain FOM, heterogeneity could be attributed more to sampling error than to differences among the FOMs (*tau*^2^ = 0.004). The undifferentiated FOM group was similarly highly heterogeneous (*I*^2^ = 85.92), and its heterogeneity was attributable more to sampling error (*tau*^2^ = 0.002). It is reasonable to argue that subgroup analysis were needed to explain the high level of heterogeneity within groups and to provide a more reliable assessment. The effect sizes did not differ statistically across categorical moderators except for the language domain.

Table 3 presents the results of the meta-regression test on the predictability of effect sizes for continuous moderator variables.

Table 3 shows that continuous variables such as study number (*β* = 0.000, *p* > .05), number of participants (*β* = 0.000, *p* > .05), and publication year of FOM (*β* = 0.006, *p* > .05) did not predict effect sizes statistically significantly. These findings indicate that the strength of the relationship between language and mathematics performance is relatively consistent across studies despite the differences in study size, temporal trends, and sample characteristics.

Table 4 presents the results of the moderator and heterogeneity analysis by the subgroups of the FOMs.

Table 4 shows that the effect sizes differed statistically significantly across the language subgroups (*Q*(b) = 9.50, *df* = 4, *p* < .05). Reading performance has a stronger relationship with mathematics performance than with other subgroups (*ES* = 0.53, 95% *CI* [0.47, 0.60]). Moreover, the relationship of reading, listening, writing, and vocabulary skills with mathematics performance is stronger than phonological performance. Yet, effect sizes did not differ statistically significantly across mathematics subgroups (*Q*(b) = 7.69, *df* = 5, *p* > .05). Likewise, effect sizes did not differ significantly across education-level subgroups (*Q*(b) = 0.19, *df* = 3, *p* > .05) and developmental pattern subgroups (*Q*(b) = 0.39, *df* = 1, *p* > .05). This implies that the relationship between language and mathematics performance is relatively consistent across different student groups and educational levels.

The primary study lists for three of the FOMs included in the dataset were unavailable. A sensitivity analysis was conducted to determine whether the effect sizes from these FOMs affected the overall effect size. Table 5 provides results of the sensitivity analysis.

This study also tested whether the effect size differed between two groups: studies with an accessible list of primary studies (with) and studies with and without an accessible list of primary studies (with and without). There was no statistically significant difference between these two groups (*Q*(b) = 0.022, *df* = 1, *p* = .64). The difference in effect size between the two groups was negligibly small (∆*ES* = 0.02). In other words, adding FOMs that were not on the list to the dataset did not statistically alter the overall effect size.

## 4. Discussion

This meta-meta-analysis probed into the relationship between students’ different language skills such as reading, writing, vocabulary, listening, and phonological processing, and their mathematics performance. After excluding several other studies for various reasons, 12 first-order meta-analyses remained for the final analysis. Considering the aggregated participant counts reported across the included first-order meta-analyses, the synthesized evidence represented more than 3,608,151 participant records. Because several primary studies were shared across first-order meta-analyses and three reviews did not report complete primary study lists, this figure reflects an aggregated rather than a unique participant count. Accordingly, the reported sample size should be interpreted as the cumulative number of participants represented across the included meta-analyses rather than the exact number of unique individuals. The effect sizes were evaluated in relation to moderator variables such as language area, mathematics area, education level, developmental pattern, publication bias, publication type, number of publications, publication year, number of participants, and location of the FOMs.

Overall, the findings point to a moderate, positive, and statistically significant relationship between language and mathematics performance. This finding aligns with the results of several prior studies that conclude that mathematics achievement significantly relies on numerical skills and also on linguistic processes (Bergqvist et al., 2018; Susperreguy et al., 2024; Ünal et al., 2021). This notable link is further supported by meta-analyses that examine the relationship between language and mathematics performance from a general perspective (Amland et al., 2025; Peng et al., 2020). Remarkably, the study offers a more comprehensive and holistic view of the relevant literature by synthesizing findings from multiple meta-analyses through a high-level synthesis approach. Taken together, these findings underline the importance of the mechanisms by which language influences mathematics performance.

Tracing the theoretical foundations of this link, one may realize that language plays a central cognitive function in structuring, representing, and making sense of mathematical knowledge (Peng et al., 2020). In fact, students need to establish a connection between verbal explanations and visual–symbolic representations to grasp mathematical knowledge (Peng & Lin, 2019; Sury & Pilchin, 2025; Swanson, 2026). On this, the Dual Coding Theory (Paivio, 1986) posits that mathematical knowledge is processed through both verbal and visual representations and that the interaction between these representations enhances learning. Studies (Fuchs et al., 2015; Peng et al., 2020; Susperreguy et al., 2024) that report a strong correlation of reading, vocabulary, and listening skills with mathematics performance support the argument that mathematical learning occurs through multiple representational systems. Working memory is where visual and verbal codes for these two different areas are processed simultaneously (Peng & Lin, 2019) and connected to each other (Swanson, 2026). Indeed, Peng et al. (2020) support these findings as they reveal that working memory plays a decisive role in both language and mathematics performance. In brief, the relationship between language and mathematics arises from basic cognitive mechanisms, such as the coordination of multiple representational systems and the effective use of working memory.

This study establishes the overall link between language and mathematics performance, but mathematics performance does not occur at the same level across all language subskills. The findings indicate that FOMs focusing on a specific language skill reported greater effect sizes than studies that dealt with language in general. This simply suggests that language is not homogeneous and that each sub-skill is linked to mathematics performance at different levels. In fact, this is also evidenced by subgroup analysis. The relationship of reading skills with mathematics performance is particularly more pronounced relative to other sub-skills. That is, reading skills are potently connected to mathematical competencies (Fuchs et al., 2015; Greisen et al., 2021; Lin & Powell, 2022; Ünal et al., 2023). The strong impact of reading skills may be explained by the fact that this skill involves not only text analysis but also higher-level cognitive processes such as making inferences, integrating information, and mentally modeling the problem situation (Lin & Powell, 2022; Sury & Pilchin, 2025). As such, reading allows the student to identify semantic–linguistic features in mathematical tasks (Boonen et al., 2016) and enables them to translate their mental representational ability into mathematical achievement (Peng et al., 2020).

Another striking finding is that the relational impact of listening, writing, and vocabulary skills on mathematical performance is greater than the impact of phonological processing skills. The low-level correlation of phonological processing skills with mathematics can be attributed to the fact that these skills are strongly linked to early language development (Hornburg et al., 2024; Whitehead et al., 2024; Yang et al., 2022) and that as the complexity of mathematical tasks increases, these skills interact with the cognitive processes required for these tasks to a more limited extent (Koponen et al., 2017; Whitehead et al., 2024). For example, the relationship of listening skills with mathematics is significantly stronger than skills such as phonological awareness or rapid automatic naming (Lu et al., 2022; Peng et al., 2020). This once again highlights that mathematics is not just about symbols but also involves an intensive linguistic process that requires processing complex instructions and verbal explanations through listening. It is worth noting that writing skills predict mathematical achievement more strongly compared to phonological processing skills, which are based only on distinguishing sounds, as they allow the student to externalize mathematical reasoning and structure abstract concepts (Kim et al., 2024). While phonological processing skills are effective in early counting and basic calculations, vocabulary plays a more critical role in deciphering the semantic structure of a problem and fostering conceptual understanding (Hornburg et al., 2024; Lin & Powell, 2022). Overall, phonological processing skills form the foundation for learning mathematics as a beginner, whereas other language skills serve as cognitive tools that entail understanding and making sense of complex mathematical tasks (Kim et al., 2024; Lin & Powell, 2022; Peng et al., 2020; Yang et al., 2022). Therefore, the stronger correlation of comprehension-based language skills with mathematics performance directly aligns with the linguistic and cognitive complexity of mathematics.

The analysis across subfields of mathematics determined that effect sizes did not differ statistically significantly depending on whether the studies were specific to a particular field of mathematics or not. Nevertheless, it is notable that many FOMs calculated effect sizes for different subfields of mathematics. However, this study did not yield a statistically significant difference between subfields. This translates to the fact that language acts as a cognitive scaffolding on mathematics performance (Peng et al., 2020; Ünal et al., 2021). Further, descriptive findings suggest that the relationship between language and verbal and applied problems tends to have a greater influence than the relationship between language and higher-order math, geometry, and arithmetic. This finding is supported by the conception that language is used not only to represent mathematical knowledge, but also as a tool that facilitates reasoning in complex tasks (Lin et al., 2021; Peng et al., 2020). Verbal problems, in particular, necessitate a higher level of language comprehension and conceptual vocabulary, unlike arithmetic fluency (Amland et al., 2025; Susperreguy et al., 2024). For this reason, while language serves as a general cognitive tool that plays a role in all subfields of mathematics, it is more influential in tasks with increased linguistic load, such as problem solving (Bergqvist et al., 2018). In this sense, focusing on improving only mental representation and arithmetic skills is insufficient for success, particularly in complex problems; focusing on reading comprehension skills is also strategically important (Boonen et al., 2016; Pongsakdi et al., 2020).

This study also finds that variables such as educational level and developmental levels did not significantly change the relationship between language and mathematics performance, which indicates that this relationship is relatively consistent across different student groups and educational levels. Such consistency indicates that the influence of language on mathematical cognition is not limited to a temporary developmental phase (He, 2016; Koponen et al., 2017; Lu et al., 2022; Peng et al., 2020; Singer & Strasser, 2017; Ulum & Küçükdanacı, 2024), but rather is integral to every stage of education and among students with varying developmental levels (Koponen et al., 2017; Lu et al., 2022; Yang et al., 2022). Still, some studies claim that this relationship varies by educational levels (Akın, 2022; Kim et al., 2024; Yang et al., 2022) and developmental levels (Akın, 2022; Peng et al., 2020). Among the studies that report differences by educational levels, Yang et al. (2022) conclude that young children need more phonological processing while acquiring basic skills such as recognizing and counting mathematical symbols; Akın (2022) puts forth that since younger students have limited reading comprehension skills, these skills are crucial for their ability to understand and solve mathematical problems. Kim et al. (2024) emphasize that the relationship between mathematics and writing is most prominent in early education and that this strong link stems from shared developmental dynamics at these ages. In terms of developmental levels, this difference is justified by the fact that students with learning disabilities experience deficiencies in language processing, and these deficiencies affect their mathematics learning more than their typically developing peers (Akın, 2022; Peng et al., 2020). The lack of a common outcome for these variables in the FOMs may be due to the differences in the primary studies examined. For example, several other factors such as affective characteristics (Wan et al., 2021), the effect of working memory (Lin & Powell, 2022; Swanson, 2026), student achievement levels (Jung, 2025), students’ different learning difficulties (Akın, 2022) or the specific subfield of mathematics under study (Sury & Pilchin, 2025) may have increased this heterogeneity. Consequently, language skills play a central role in early mathematics development. However, its importance in the later stages of education should not be underestimated. Likewise, for developmental levels, language skills are crucial for mathematics performance, regardless of whether the student has a learning disability. Overall, the relationship between language and mathematics performance should be viewed not as a limited interaction occurring only in specific student profiles, but rather as a core academic relationship shaped by cognitive, developmental, and contextual variables, yet remaining consistent across different educational levels.

The high level of heterogeneity in the study suggests that the relationship between language and mathematics performance may vary depending on contextual factors. There are several other FOMs that report a high level of heterogeneity (Akın, 2022; He, 2016; Kim et al., 2024; Yang et al., 2022). The reason for such variation may be complex variables such as the language of instruction, teaching methods, the nature of the measurement tools used, and the socio-economic level, which cannot be assessed within this study. Consistent with this, Bergqvist et al. (2018) concluded that mathematics achievement depends not only on mathematical ability but also on the structural characteristics of the language used, and that this effect varies from language to language. Regarding teaching methods, Arsenault et al. (2025) revealed that the positive effect of mathematics writing instruction on mathematics achievement is more pronounced, especially in interventions focusing on the informative type of writing and structured discussion processes. In sum, the link between language and mathematics should be considered not as a fixed property within a single context, but as a multidimensional property sensitive to different contexts.

## 5. Limitations

This research has some limitations, especially from a methodological point of view. First, the lack of primary studies in three of the FOMs included in the study made it challenging to properly assess the overlap among the FOMs. Although the overall overlap was found to be low and sensitivity analysis were performed to test this, potential data duplication cannot be entirely ruled out. As a result of the sensitivity analysis, we observed that the model was not sensitive to FOMs that were not on the list. It can be said that including or excluding these studies did not affect the calculated effect sizes. However, the analysis was conducted on the assumption that the three FOM studies not on the list did not overlap significantly with the other FOMs. These FOMs may or may not overlap. In this study, unlisted studies were included to ensure that no unique foundational research was overlooked. In this context, it can be said that the inability to fully validate the overlap analysis falls within the limitations of this study. Second, this study detected no publication bias, and the effect sizes did not differ by the presence of publication bias. Yet, it is worth noting that the lack of reports on publication bias in some of the examined FOMs represents another limitation. Third, there is insufficient evidence on the relationship between subfields of mathematics and language. While studies offer clear evidence on the individual examination of language skills, they tend to consider mathematics as a whole; meta-analyses focusing on subfields such as arithmetic, geometry, and algebra are few in number. Moreover, the limited data on the linguistic and cultural contexts in which studies were conducted make it difficult to thoroughly examine the impact of language’s structural features on mathematics performance. All of these limit the statistical power and generalizability of this study’s findings. For this reason, the findings from some moderator analysis require careful interpretation.

## 6. Conclusions and Implications

This meta-meta-analysis finds a moderate and positive relationship between language and mathematics performance. The reliability of its findings is further supported by the absence of significant publication bias in the publication bias analysis and the increased stability of the findings indicated by the sensitivity analysis. Taken together, the observed effect demonstrates that language skills play not only an instrumental but also a significant and systematic role in mathematics performance. In this respect, the study not only confirms the findings in the relevant literature about the relationship between language and mathematics but also provides deeper insights into the scope and nature of this relationship. By combining the findings of various meta-analyses within a holistic framework, this study addresses the fragmented structure of the literature and offers a broader conclusion about the relationship between language and mathematics. Moreover, this study concludes that language skills such as reading and listening comprehension have a stronger relationship with mathematics performance, which suggests that mathematical thinking is significantly supported by language-based cognitive processes. Yet another striking finding is that language does not have a homogeneous nature; rather, each specific language skill examined has a unique and profound correlation with mathematics performance. The consistency of this relationship across different ages and education levels confirms that language–mathematics interaction is of critical significance at all stages of academic development. In conclusion, this study presents strong empirical evidence that the relationship between language and mathematics is a vital component of mathematics learning, rather than a contextual or limited interaction, and that these two areas should be addressed together.

In light of the findings, the researchers propose that a holistic approach should be adopted to mathematics instruction, incorporating practices such as reading and listening comprehension, vocabulary, and the effective use of mathematical language in writing and speech. To achieve this, it is essential to design mathematics curricula, textbooks, and teaching materials with a holistic approach that considers the interaction between language and mathematics. These materials would enhance students’ understanding of mathematical concepts. Also, instructors need to place greater emphasis on linguistic explanations, the meaning of concepts, and the understanding of problem texts in mathematics lessons. Such changes in instruction would help acknowledge the relationship between language and mathematics at all grade levels. Further, longitudinal and experimental research designs would allow scholars to have a deeper understanding of this relationship. Similarly, comparative studies examining the impact of typological features of languages on mathematics learning would offer valuable insights into the cultural and linguistic dimensions of this relationship. Scholars may also conduct primary meta-analyses specific to subfields of mathematics (e.g., arithmetic, algebra, and geometry) to reveal how the language–mathematics relationship differs across fields, thereby allowing stronger generalizations of findings. Lastly, the current literature seems to focus mainly on cognitive performance. More scholarly attention is needed to address the affective aspects of the relationship between language and mathematics (e.g., math anxiety, attitudes, and self-efficacy). Exploring these aspects would help fill the gaps in the relevant literature.

## Figures and Tables

**Table 1:** Inclusion and Exclusion Criteria.

Criteria	Inclusion	Exclusion
Outcome	Accordingly, any FOM research was included if they focused on Language and mathematics related to performance aspects such as knowledge, achievement, and skills.	FOMs focusing on clinical outcomes specific to the brain and neurological fields were excluded.
Participants	FOM research that involved only students as their participants was included. Studies can involve students at all levels of education, from kindergarten through higher education, including students with typical or atypical developmental levels.	Meta-analyses involving adult samples were excluded. Further, studies with immigrant students learning mathematics as a second language and multilingual students were not included either.
Study design	Regarding study design, eligible FOMs were required to be correlational and conducted within a survey model.	FOMs examining the impact of intervention programs were excluded. The effect sizes of FOMs that synthesize the effect of intervention programs may be related to different moderator variables.
Data availability	Eligible FOMs must provide sufficient statistical values for the generic effect size calculation.	FOMs lacking the necessary statistical data for the generic effect size extraction were excluded.
Language and publication status	Only FOMs written in English were considered eligible for inclusion; in terms of publication type, eligibility was limited to published articles, academic papers, reports, and doctoral dissertations.	Non-English FOMs were excluded.
Overlapping	Eligible FOMs should be independent of one another	A corrected covered area (CCA) between FOMs that exceeds 10% signifies a high level of overlap (Pérez-Bracchiglione et al., 2022). The study with the narrowest scope among overlapping FOMs was excluded.

**Table 2:** Categorical Moderators and Heterogeneity Analysis.

Group	*k*	*ES*(*Fz*)	*LL*	*UL*	*Z*	*p*	*Q*(t)	*p*	*I* ^2^	*tau* ^2^
Language domain										
Specific language domain	8	0.55	0.50	0.59	25.28	<.001	82.10	<.001	91.47	0.004
Undifferentiated	4	0.43	0.37	0.49	14.26	<.001	21.31	<.001	85.92	0.002
*Q*(b) = 10.05, *df* = 1, *p* = .002										
Math domain										
Specific math domain	1	0.62	0.46	0.78	7.60	<.001				
Undifferentiated	11	0.50	0.45	0.55	19.32	<.001	211.51	<.001	95.27	0.006
*Q*(b) = 2.01, *df* = 1, *p* = .16										
Education scope of FOM										
Pre/K12	10	0.50	0.44	0.56	17.06	<.001	244.84	<.001	96.32	0.008
Pre/K12 and Higher	2	0.55	0.43	0.67	8.76	<.001	4.97	.03	79.86	0.001
*Q*(b) = 0.52, *df* = 1, *p* = .47										
Presence of publication bias										
Minimal	2	0.42	0.29	0.55	6.14	<.001	5.32	.02	81.19	0.001
Not reported/Insufficient	3	0.52	0.41	0.63	9.12	<.001	9.01	.01	77.80	0.002
Substantial publication bias	2	0.55	0.42	0.68	8.08	<.001	4.97	.03	79.86	0.001
Unidentified	5	0.53	0.44	0.62	11.46	<.001	182.38	<.001	97.81	0.016
*Q*(b) = 2.28, *df* = 3, *p* = .52										
Participant in FOM										
Atypically developing	1	0.58	0.40	0.75	6.39	<.001				
Undifferentiated	11	0.50	0.45	0.56	17.93	<.001	268.95	<.001	96.28	0.008
*Q*(b) = 0.61, *df* = 1, *p* = .44										
Location in FOM										
Chinese	1	0.38	0.24	0.52	5.34	<.001				
Multi-country	11	0.52	0.48	0.57	22.96	<.001	152.04	<.001	93.42	0.005
*Q*(b) = 3.75, *df* = 1, *p* = .05										
Publication type of FOM										
Article	11	0.52	0.46	0.57	18.58	<.001	281.77	<.001	96.45	0.008
Doctoral dissertation	1	0.44	0.25	0.63	4.47	<.001				
*Q*(b) = 0.60, *df* = 1, *p* = .44										

**Table 3:** Results of the Meta-Regression Test for Continuous Variables.

Covariate	*β*	*SE*	*LL*	*UL*	*Z*	*p*
Intercept	0.503	0.036	0.432	0.575	13.83	<.001
Study number	0.000	0.000	−0.000	0.001	0.21	.830
*Q*(model) = 0.05, *df* = 1, *p* = .83						
Intercept	−11.128	19.178	−48.716	26.460	−0.58	.562
Year of FOM	0.006	0.010	−0.013	0.024	0.61	.544
*Q*(model) = 0.37, *df* = 1, *p* = .54						
Intercept	0.488	0.038	0.413	0.562	12.82	<.001
*N* (participant)	0.000	0.000	0.000	0.000	0.85	.394
*Q*(model) = 0.73, *df* = 1, *p* = .39						

**Table 4:** Sub-group Moderators and Heterogeneity Analysis.

Subgroups	*k*	*ES*(*Fz*)	*LL*	*UL*	*Z*	*p*	*Q*(t)	*p*	*I* ^2^	*tau* ^2^
Subgroup of math										
Algebra	3	0.48	0.35	0.61	7.13	<.001	3.90	.142	48.74	0.008
Applied problems	1	0.52	0.30	0.74	4.69	<.001				
Arithmetic	12	0.40	0.36	0.45	17.69	<.001	134.94	<.001	91.85	0.005
Geometry	3	0.38	0.26	0.50	6.04	<.001	0.20	.905	0.00	0.000
Higher order math	2	0.34	0.23	0.46	5.93	<.001	2.26	.133	55.70	0.002
Word problems	5	0.49	0.42	0.56	12.93	<.001	25.67	<.001	84.41	0.006
*Q*(b) = 7.69, *df* = 5, *p* = .17										
Subgroup of language										
Listening comprehension	2	0.51	0.39	0.64	7.94	<.001	6.05	.014	83.47	0.006
Phonological performance	4	0.37	0.28	0.46	8.26	<.001	16.70	<.001	82.03	0.002
Reading performance	8	0.53	0.47	0.60	16.59	<.001	260.20	<.001	97.31	0.011
Vocabulary	3	0.45	0.36	0.55	9.10	<.001	58.80	<.001	96.60	0.004
Writing performance	2	0.48	0.35	0.60	7.48	<.001	5.93	.015	83.15	0.004
*Q*(b) = 9.50, *df* = 4, *p* = .04										
Developing type										
Atypically developing	3	0.52	0.42	0.63	9.73	<.001	68.10	.001	97.06	0.020
Typically developing	3	0.48	0.38	0.58	9.18	<.001	8.56	.014	76.63	0.001
*Q*(b) = 0.39, *df* = 1, *p* = .53										
Education level										
Kindergarten	3	0.43	0.26	0.61	4.86	<.001	15.04	.001	86.71	0.040
Elementary	7	0.47	0.36	0.58	8.05	<.001	54.94	.001	89.08	0.015
Secondary	3	0.49	0.29	0.68	4.89	<.001	5.09	.079	60.68	0.014
College	1	0.45	0.10	0.80	2.49	.01				
*Q*(b) = 0.19, *df* = 3, *p* = .98										

**Table 5:** Sensitivity Analysis of Dataset.

Group	*k*	*ES*(*Fz*)	*LL*	*UL*
With list	9	0.49	0.43	0.56
With and without list	12	0.51	0.46	0.56
*Q*(b) = 0.022 *df* = 1 *p* = .64				

## Data Availability

Data and analysis code for this study are available at https://osf.io/kz7yt/overview (accessed on 1 June 2026).

## Supplementary Materials

The following supporting information can be downloaded at: https://www.mdpi.com/article/10.3390/jintelligence14080152/s1, Table S1: Prisma checklist.
